# In young children, persistent wheezing is associated with bronchial bacterial infection: a retrospective analysis

**DOI:** 10.1186/1471-2431-12-83

**Published:** 2012-06-22

**Authors:** Iris De Schutter, Alexandra Dreesman, Oriane Soetens, Marc De Waele, Françoise Crokaert, Jan Verhaegen, Denis Piérard, Anne Malfroot

**Affiliations:** 1 Department of Pediatric Pulmonology, CF-Clinic and Pediatric Infectious Diseases, Universitair Ziekenhuis Brussel (UZ Brussel), Brussels, Belgium; 2 Department of Microbiology and Infection Control, Universitair Ziekenhuis Brussel (UZ Brussel), Brussels, Belgium; 3 Department of Hematology, Universitair Ziekenhuis Brussel (UZ Brussel), Brussels, Belgium; 4 Department of Microbiology, CHU St. Pierre, Brussels, Belgium; 5 Department of Microbiology, UZ, Gasthuisberg, Leuven, Belgium

**Keywords:** Preschool wheezing, Bronchial bacterial infection, Neutrophilic inflammation, Non-Typeable *Haemophilus Influenzae*

## Abstract

**Background:**

Young children with persistent wheezing pose a diagnostic and therapeutical challenge to the pediatrician.

We aimed to evaluate bacterial bronchial infection as a possible reason for non response to conventional asthma therapy, and to identify and characterise the predominant pathogens involved.

**Methods:**

We retrospectively analysed microbiological and cytological findings in a selected population of young wheezers with symptoms unresponsive to inhaled corticosteroid (ICS) therapy, who underwent flexible bronchoscopy with bronchoalveolar lavage (BAL). Procedural measures were taken to limit contamination risk and quantitative bacterial culture of BAL fluid (significance cut-off ≥ 10^4^ colony-forming units/ml) was used. Modern microbiological methods were used for detection of a wide panel of pathogens and for characterisation of the bacterial isolates.

**Results:**

33 children aged between 4 and 38 months, without structural anomalies of the conductive airways were evaluated. Significant bacterial BAL cultures were found in 48,5 % of patients. *Haemophilus influenzae* was isolated in 30,3 %, *Streptococcus pneumoniae* in 12,1 % and *Moraxella catarrhalis* in 12,1 %. All *H. influenzae* isolates were non-encapsulated strains and definitely distinguished from non-haemolytic *H. haemolyticus*. Respiratory viruses were detected in 21,9 % of cases with mixed bacterial-viral infection in 12,1 %. Cytology revealed a marked neutrophilic inflammation.

**Conclusions:**

Bacterial infection of the bronchial tree is common in persistent preschool wheezers and provides a possible explanation for non response to ICS therapy. Non-typeable *H. influenzae* seems to be the predominant pathogen involved, followed by *S. pneumoniae* and *M. catarrhalis.*

## Background

Recurrent and/or persistent wheezing in young children is a major reason for use of pediatric healthcare resources [1,2]. Despite the high morbidity of persistent infantile wheezing, there are only few comprehensive studies in this domain, resulting in poor knowledge of the pathophysiology and a lack of efficacy data of therapeutical interventions [3]. Although 3 pediatric asthma phenotypes have been described, in daily practice preschool wheezers remain a diagnostic and therapeutic challenge to the pediatrician [4,5].

Current asthma guidelines, recommend a trial with inhaled corticosteroids (ICS) in these children, based on the established pathophysiology of eosinophilic inflammation and the proven efficacy in asthma in adults [2,6,7].

From clinical experience and scientific reports, it is apparent that preschool wheezers are a heterogeneous group of which only part will benefit from the use of ICS [8-10]. Several authors recommend an extensive work-up in ICS non-responders, including gastro-oesophageal reflux and allergy testing, sweat chloride test and bronchoscopy with bronchoalveolar lavage (FOB + BAL) to evaluate for underlying structural anomalies, comorbidities, underlying genetic disorders and an infectious etiology [2,5,6,9,10].

In this report, we describe the findings of FOB + BAL, in a selected group of young persistent wheezers without underlying structural anomalies. We focus on the microbiological findings and discuss the possible pathophysiology of bronchial bacterial infection.

## Methods

### Patients

Children with physician diagnosed, persistent wheezing unresponsive to ICS therapy, who underwent FOB + BAL between January 2005 and December 2007 were included. As a tertiary referral centre, we are referred children for further evaluation and treatment of persistent wheezing despite ICS treatment and anti-reflux therapy in those with demonstrated GERD. Our initial assessment includes a detailed history of disease, symptoms, signs and treatment adherence, a check for the appropriateness of treatment administration and dosage, and a thorough physical examination. In children without any apparent explanation for persistent wheezing, a work-up, including FOB + BAL is conducted. In these cases, FOB + BAL is always performed under stable condition, outside episodes of acute infection or exacerbation of respiratory symptoms.

Children with symptoms other than wheezing, such as chronic wet cough, recurrent bronchitis and stridor, being the main reason for FOB + BAL were excluded. Children with respiratory tract anomalies including malacia of the conductive airways, or with known underlying chronic conditions, such as cystic fibrosis, primary ciliary dyskinesia, immune deficiency, bronchopulmonary dysplasia, congenital heart diseases and neuromuscular disorders were also excluded.

When available, results of allergy and reflux testing, data on anti-reflux therapy prior to FOB + BAL, familial history of allergy and asthma, a history of exposure to tobacco smoke, and the response to antibiotic treatment were recorded. Children with positive skin prick tests and/or elevated specific immunoglobulin E in blood were considered allergic. GERD was assessed by a positive 24 hour-oesophageal pH measurement, interpreted according to gastroenterological criteria [11].

Flexible fibroscopic bronchoscopy with bronchoalveolar lavage:

FOB and BAL-sampling were performed according to European Respiratory Society recommendations, as previously described [12,13]. BAL-samples were divided into 2 aliquots, which were immediately sent to the Microbiology lab and the Hematology lab.

### Laboratory methods

#### Microbiology

Upon arrival in the lab, BAL-samples were processed for smear, bacterial culture, viral culture, and for the detection of influenzavirus A and B; parainfluenzavirus types 1, 2, 3; RSV; human metapneumovirus subtypes A and B; coronaviruses 229E and OC43, *Mycoplasma pneumoniae* and *Chlamydophila pneumoniae* using an in-house multiplex Polymerase Chain Reaction (mPCR)- protocol, as described previously [13]. The performance of this test was assessed by regular participation in the external quality programme organised by QCMD (West of Scotland Science Park Glasgow G20 0XA Scotland, UK).

Gram staining of smear and bacterial BAL-culture were performed according to standard methods [14,15]. 70 μL of BAL-fluid was centrifugated on a cytospin slide and Gram stained. Samples were considered representative of the lower airways, if >40 % of instilled volume was recovered and if < 1 % of nucleated cells are squamous epithelial cells on smear. Undiluted and 1 μL of a 1/1000 dilution of BAL-fluid were inoculated on blood agar supplemented with X and V factors incubated at 35 °C in 5 % CO_2_, MacConkey agar and mannitol salt agar incubated at 35 °C in air. Growth was assessed after 24 h, and after 48 h in case of no growth at 24 h. Results were reported in semi-quantitative terms, with a growth of potential pathogens of ≥10^4^ cfu/ml considered significant.

All *H. influenzae* and *S. pneumoniae* isolates were sent to the respective Belgian reference lab for capsular typing as described elsewhere [13].

All NTHi isolates were discriminated from non-haemolytic *Haemophilus haemolyticus* (non-haemolytic *H. haemolyticus)* using molecular techniques [13].

#### Cytology

BAL-fluid was transported to the laboratory in a siliconized container on ice. The fluid was filtered through nylon gauze. After staining with brilliant cresyl blue, total nuclear cell counts were performed in a Bürker counting chamber under light microscopy at a magnification of 250x. A cell suspension of 2.10^6^ cells/ μl was made in phosphate-buffered saline with 1 % bovine serum albumin (PBS-BSA). Cytocentrifuge preparations were made and stained by May-Grünwald Giemsa. Two hundred cells were examined for differential cell counts.

Given that the median percentage of neutrophils in BAL-fluid of healthy children is 0.9-3.5 %, we chose to base our definition for neutrophilic inflammation in BAL-fluid on presence of >10 % neutrophils in the cytological differentiation, as previously done by Saito et al. [10,12].

### Statistics

Statistical analyses were performed using SPSS IBM Statistics version 19 software. Mann-Withney Test was used for equality of medians, with a 2-tailed p-value < 0.05 considered statistically significant. Multiple comparisons were handled by the Holm's method.

### Ethics

In all cases FOB + BAL was medically indicated. Full parental consent was obtained orally after explanation of the procedure, its benefits and its risks.

Retrospective chart analysis was approved by the ethical committee of the Universitair Ziekenhuis Brussel (UZ Brussel).

## Results

Thirty-three children, 12 girls and 21 boys with a median age of 10 months (range 4-38 m), were assessed because of unexplained persistent wheezing despite ICS therapy. Administrated ICS dosages ranged from 100–500 mcg fluticasone daily or equivalent, with a variable treatment duration of 1–10 months.

Table 1 summarises the characteristics of the study population with respect to allergy and reflux testing results, and familial and environmental risk factors for asthma. Only 7 of 13 (53.8 %) children suffering from gastro-oesophageal reflux disease (GERD), were treated for GERD prior to FOB + BAL.

**Table 1:** Patients characteristics: comorbidities, and familial and environmental risk factors for asthma

**Characteristic n = 33**	**Yes*******n******* (%)**	**No*****n***** (%)**	**Unknown*****n***** (%)**
**allergy**	5 (15.2)	24 (72.7)	4 (12.1 )
**GERD**	13 (39.4)	15 (45.5)	5 (15.2 )
**allergy/ asthma in first degree relatives**	14 (42.4)	12 (36.4)	7 (21.2)
**tobacco exposure**	3 (9.1)	16 (48.5)	14 (42.4)

None of the children were treated with antibiotics in the 7 days prior to FOB + BAL.

The FOB + BAL procedure was well tolerated in all children.

Significant bacterial cultures were found in 16 of 33 (48.5 %) children (Table 2). No significant differences in the number of positive BAL-cultures were seen in children with or without GERD.

**Table 2:** Overview of microbiological results

	**Mono- culture*****n***** (%)**	**Co-infection*****n***** (%)**	**Total*****n***** (%)**
**Significant BAL-culture**	**11/33 (33.3)**	**5/33 (15.2)**	**16/33 (48.5)**
*Haemophilus influenzae*	6 (18.2)^e^	4 (12.1)^a^	10 (30.3)
*Streptococcus pneumoniae*	1 (3.0)^d^	3 (9.1)	4 (12.1)
*Moraxella catarrhalis*	2 (6.1)	2 (6.1)^a^	4 (12.1)
*Staphylococcus aureus*	0	1 (3.0)^c^	1 (3.0)
*Escherichia coli*	2 (6.1)^b^	0	2 (6.1)
*Serratia marcescens*	0	1 (3.0)^c^	1 (3.0)
**Commensal flora**			**15 (45.4)**
**< 10**^**4**^ **cfu/ml potential pathogens in BAL**			**2 (6.1)**
**Viral infection*n*(%)**	**5/32 (15.6)**	**2/32 (6.3)**	**7/32 (21.9)**
Respiratory syncytial virus	2 ^a^	1^b^	3
Adenovirus	0	1^b^	1
Cytomegalovirus	1	0	1
Enterovirus (not polio)	0	1^c^	1
Coronavirus E229	1^d^	0	1
Human metapneumovirus	1	0	1
Parainfluenzavirus type 3	0	1^c^	1
**No viral infection detected*n*(%)**			**25/32 (78.1)**
**Atypical microorganism**	**positive**	**negative**	**Total**
*Mycoplasma pneumoniae*	1^e^	18	1/19
*Chlamydophila pneumoniae*	0	14/14	0/14

Co-infection: cases in which at least 2 infective agents of the same microbiological group were isolated.

^a,b,c,d,e^ Mixed infections: cases in which at least 2 infective agents of a different microbiological group were detected. Mixed infections are mentioned for each pathogen.

Aerobic bacteria were isolated as sole pathogen in 11 of 33 (33.3 %) cases. Mixed bacterial-viral infection and mixed bacterial-atypical infection were found in 4 of 33 (12.1 %) and in 1 of 33 (3.0 %) cases, respectively (Table 2).

Considering only significant bacterial BAL-cultures, *Haemophilus influenzae* (*H. influenzae*), isolated from 10 of 33 (30.3 %) children, was the predominant bacteria found, followed by *Streptococcus pneumoniae* (*S. pneumoniae*) and *Moraxella catarrhalis* (*M. catarrhalis*) (Table 2). Bacterial co-infection was found in 5 of 33 (15.2 %) cases, including *H. influenzae* in 4 of these; twice with *S. pneumoniae*, once with *M. catarrhalis* and once with both *S. pneumoniae* and *M. catarrhalis,* and 1 co-infection with *Staphylococcus aureus* and *Serratia marcescens* (Table 2).

Respiratory viruses were detected in 7 of 32 (21.9 %) cases, with predominance of respiratory syncytial virus (RSV) (Table 2). In 3 of 32 (9.4 %) cases the respiratory virus was the sole pathogen detected (Table 2). *Mycoplasma pneumoniae* was detected in 1 of 19 cases, in a mixed infection with *H. influenzae* (Table 2).

Capsular typing of *H. influenzae* isolates revealed that all 10 isolates were non-encapsulated strains (NTHi). All 10 NTHi strains were evaluated for misidentification of non-haemolytic *H. haemolyticus*, and all were proven true NTHi. Nine of ten (90 %) NTHI isolates were β-lactamase negative and susceptible to ampicillin (MIC ≤ 1μg/ml) and cefuroxime (MIC ≤ 1μg/ml) [16]. The latter isolate was β-lactamase positive, with a MIC = 2μg/ml for amoxicillin-clavulanate and MIC = 3μg/ml for cefuroxime.

The 4 *S. pneumoniae* isolates were fully susceptible to penicillin and belonged to serogroups; ST 6, ST 9, ST 11 and ST 19.

Susceptibility based antibiotic treatment in patients with a significant BAL-culture is routine in our centre. In general, antibiotics are administered orally for 10 days (daily dose: 50–75 mg/kg for amoxicillin or amoxicillin-clavulanate). 7 Of 16 (43,8 %) patients responded well to treatment, in 3 of 16 (18,8 %) no change in symptoms was noted, and data were missing for 6 patients. In 4 of 7 (57,1 %) responders however, symptoms relapsed within 2–3 weeks after completion of the antibiotic treatment. In one of these patients, FOB + BAL was repeated 70 days after the initial procedure. NTHi (>10^5^ colony forming units (cfu)/ml) that was present as sole pathogen in the first BAL fluid was cultured again together with *S. pneumoniae* (>10^5^ cfu/ml).

The Total cell count (TCC) in BAL-fluid was high (median: 1055.5/mm^3^, IQR: 544.5/mm^3^ - 2099.75/mm^3^) and differential cytology revealed a neutrophilic inflammation in 24 of 28 (85.7 %) patients.

Patients with a positive bacterial BAL-culture, had a significantly higher TTC (median 1893.5/mm^3^, IQR 829.7/mm^3^- 3479.5/mm^3^ versus 787.0/mm^3^, IQR 283.5/mm^3^ - 1439.5/mm^3^; p = 0.023) compared to patients with a negative BAL-culture. Cytology of BAL-fluid differed significantly between patients with NTHi isolated from BAL and patients with a negative BAL-culture (Table 3).

**Table 3:** Cytology of BAL in patients with NTHI-isolated from BAL compared to patients with negative BAL-culture

	**BAL-culture: NTHi**	**BAL-culture: negative**	**p-value**
**Total cell count** (/mm^3^)	2913.5 (1307.0-6460.0)	827.0 (344.5-1506.2)	**.003**
**Neutrophils**			
cells/mm^3^	2179.0 (318.1-5306.6)	162.3 (63.7-448.8)	**.014**
%	71.5 (28.2-85.6)	24.5 (14.0-47.5)	NS^a^
**Lymphocytes**			
cells/mm^3^	153.6 (19.6-345.8)	19.7 (2.6-88.5)	.06
%	5.5 (3.4-9.0)	6.0 (2.0-10.0)	NS^a^
**Eosinophils**			
cells/mm^3^	0.0 (0.0-0.0)	0.0 (0.0-0.65)	NS
%	0.0 (0.0-0.0)	0.0 (0.0-0.5)	NS^a^
Alveolar macrophages*			
cells/mm^3^	547.5 (115.0-891.2)	409.0 (207.7-653.8)	NS
%	22.5 (8.25-44.0)	73.5 (48.0-78.0)	**.008**^**a**^

Values are expressed as median (IQR).

* with epithelial cells included.

^a^ adjusted p-values for multiple comparisons by Holm's Method.

## Discussion

In contrast to viral infections, which are known to be associated with wheezing in young children, bacterial infections have long been considered of no importance in asthma and wheezing, and antibiotic therapy is not recommended in current asthma treatment guidelines [17-20]. However, several studies suggest that bacteria might play a role in wheezing in young children [9,10,17,19,20]. Moreover, it is increasingly appreciated that the human body is home to an extended microbiome, with asthmatic children harbouring different bacteria and a less diverse mix compared to healthy children [21]. Characterisation of the predominant bacteria in wheezing children is essential to understand the pathophysiology of persistent wheezing.

Recently, tracheobronchomalacia has been shown to be highly associated with bronchial inflammation and bacterial infection in children with persistent respiratory symptoms [22]. Nevertheless, it remains unclear whether bacterial infection is simply a consequence of impaired mucociliary clearance or whether it is an independent inflammatory stimulus in persistent wheezers.

We hypothesized that bacterial infection might indeed be an independent inflammatory stimulus in persistent preschool wheezers, and chose to study a selected group of patients without structural anomalies of the conductive airways.

In our study, we found that bronchial bacterial infection is common in persistent preschool wheezers without apparent reasons for impaired mucociliary clearance, and that *H .influenzae* was the predominant aerobic pathogen isolated, followed by *S. pneumoniae* and *M. catarrhalis.* We also found frequent bacterial co-infection involving these 3 pathogens. Our findings corroborate observations of other authors, who also described the predominance of this bacterial triad in children with persistent or recurrent wheezing [9,10,19,20,23,24].

The frequent upper respiratory tract colonisation with these aerobic bacteria in children, may provide difficulties to discriminate between contamination and true bronchial infection [25]. However, we consider our results to reflect true bronchial infection, as we applied procedural methods, which were shown to limit strongly the risk for contamination [13,20,26,27]. In particular, we chose to use a cut-off of ≥10^4^ cfu/ml for bacterial BAL-culture, although in adults a cut-off of ≥10^3^ cfu/ml has demonstrated to be discriminative [28,29]. The applied cut-off was also used previously by others, studying children with chronic bronchitis [30]. Moreover, although FOB + BAL were only performed once in the majority of patients, our results probably reflect chronic bronchial infection as all our samples were collected during stable clinical state.

The presence of a significant increased neutrophilic inflammation in BAL-fluid of those patients in which NTHi was cultured, as compared to patients with a negative BAL-culture, supports the hypothesis that bacterial infection is indeed an inflammatory stimulus in children with persistent wheezing[24].

*H. influenzae* was the predominant aerobic bacterial pathogen, isolated in 30.3 % of our patients. Although previously other authors have had similar results, isolated strains of *H. influenzae* were never further characterised [9,10,20,23]. To our knowledge this is the first report that associates bronchial infection with non-typeable *H. influenzae* (NTHi) in young persistant wheezers. Identification of NTHi as the predominant pathogen further supports our hypothesis that persistent wheezers present an underlying inflammation induced by chronic bacterial infection. Chronic NTHI infection, resulting from the ability of NTHI to persist in the lower airways through several escape mechanisms from the host immune responses, has previously been reported by others [31-33]. Moreover, in the lower airways of patients with chronic obstructive lung disease, NTHI has been demonstrated to be a major inflammatory stimulus [31,34].

The poor response of symptoms to an in-vitro susceptibility guided, adequately dosed antibiotic treatment that we found, is similar to poor response findings in 2 other disease entities involving *H. influenzae* as a predominant pathogen in children; recurrent and unresponsive otitis media and protracted bacterial bronchitis [35-38]. In the middle ear, biofilm formation has been demonstrated to be one of the major mechanisms for persistence and provides an explanation for unresponsiveness to antibiotic therapy [39,40]. Moreover, biofilm formation and intracellular infection are well established mechanisms of NTHi for persistence in the lower airways [31-33]. Although our study does not provide evidence of biofilm formation in the lower airways of young unresponsive wheezers, our findings of poor response to and frequent relapse after a short course of antibiotic treatment are suggestive for involvement of biofilm. Biofilm formation should be addressed in future studies, because its presence may have consequences for treatment.

The strength of our study is the exhaustive microbiological evaluation with use of semi-quantitative BAL-cultures, multiplex-PCR (mPCR) for viral and atypical agents, and thorough characterisation of the isolated *H. influenzae* strains. However, we may have missed some cases of viral infection because human rhinovirus was not included in the applied PCR-protocol.

The major weakness of our study is its retrospective character and the absence of an age-matched control group. However, reports, from prospective conducted studies without profound characterisation of the isolates, describing association between wheezing and bacterial infection support our findings [10,19,22]. The absence of a control group is considered a minor weakness, as previous publications have demonstrated the limited risk for contamination of BAL when technical precautions are taken [13,20,26,27]. Moreover, performance of FOB + BAL is considered unethical in healthy children [10,12].

Another limitation is the small patient cohort. Due to the multiple exclusion criteria applied, the patients included in the analysis represented only 1,2 % of children seen in our clinic because of persistent respiratory symptoms. Nevertheless, analysis of this highly selected population without structural causes of impaired mucus clearance, enabled us to evaluate the association between bacterial bronchial infection, neutrophilic inflammation and symptoms resistant to ICS therapy.

## Conclusions

In preschool children with persistent wheezing unresponsive to adequate inhaled asthma treatment, bacterial infection is frequent, with NTHi, *S. pneumoniae* and *M. catarrhalis* being the main pathogens involved. These microbiological findings and the presence of a pronounced neutrophilic inflammation in BAL-fluid, sampled in patients not presenting an acute exacerbation episode, favours the hypothesis that chronic bacterial infection is an ongoing inflammatory stimulus providing a possible explanation for persistent wheezing despite ICS therapy in these young patients. These elements suggest that the use of antibiotic treatment, although not currently recommended, should be considered in selected young children presenting with chronic wheezing. However, as these patients are indistinguishable without the use of invasive procedures, and biofilm formation and other escape mechanisms may decrease the bactericidal efficacy of antibiotic treatment, additional research is necessary before specific treatment recommendations can be provided. Future research should focus on population characteristics, determination of risk factors for acquisition of bacterial bronchial infection, pathogenicity mechanisms of chronic bronchial infection and possible prevention and treatment strategies.

## Abbreviations

BAL, BronchoAlveolar Lavage; CFU, Colony-Forming Unit; FOB, Flexible Fibroscopic Bronchoscopy; GERD, Gastro-Oesophageal Reflux Disease; ICS, Inhaled CorticoSteroid; mPCR, Multiplex Polymerase Chain Reaction; NTHi, Non-Typeable Haemophilus Influenzae; TCC, Total Cell Count.

## Competing interests

AM and IDS have participated in advisory boards on pneumococcal diseases and vaccines for Wyeth-Pfizer and GSK. IDS has participated in advisory boards on NTHi for GSK. AM, IDS and JV are member of a steering committee of a research project sponsored by Wyeth-Pfizer and AM is coordinating a steering committee of a research project sponsored by Wyeth-Pfizer. AM has been a speaker for Wyeth-Pfizer and/ or GSK on pneumococcal diseases and epidemiology. IDS has been a speaker for Pfizer on pneumococcal pneumonia. JV is heading the national reference laboratory for *S. pneumoniae* that received grants from Wyeth-Pfizer and GSK for serotyping isolates of IPD studies. DP received travel grants from Janssen-Cilag, Wyeth and Pfizer and support for a research project concerning antibiotic susceptibility of anaerobes from Pfizer, Astra-Zeneca and Bayer. All other authors declared no conflicts of interest.

## Authors’ contributions

All authors substantially contributed to the study, with regards to design, conduction, analysis of the results and/or manuscript preparation. IDS, AD and AM have contributed to all the aspects of the work: conception and design, data retrieval and analysis, and preparation of the manuscript. OS, MDW, FC, JV and DP have contributed to acquisition and analysis of the data, and have critically reviewed the manuscript. All co-authors have approved the final version of the manuscript.

## Funding

This study was supported by an unrestricted scientific grant from GSK Biologicals. The grant enabled us to engage an independent data nurse for retrieval of the microbiological data and database-cleaning.

## Pre-publication history

The pre-publication history for this paper can be accessed here:

http://www.biomedcentral.com/1471-2431/12/83/prepub
